# From symptom relief to household control: baloxavir redefines antiviral strategies for influenza

**DOI:** 10.1016/j.lanwpc.2025.101651

**Published:** 2025-08-02

**Authors:** Jin-Fu Xu, Yang Liu, Panpan Zhang, Bin Cao

**Affiliations:** a Department of Respiratory and Critical Care Medicine, Huadong Hospital, Fudan University, Shanghai, 200040, China; b Department of Pulmonary and Critical Care Medicine, China-Japan Friendship Hospital, Capital Medical University, Beijing, 100054, China

The global burden of influenza remains heavy, with an estimated 650,000 annual deaths worldwide and an average of 88,000 annual deaths in China, resulting in significant economic costs from healthcare utilization and productivity loss.^1^^,^^2^ Household transmission plays a pivotal role in the spread of influenza virus, accounting for a large proportion of cases, with secondary attack rates reaching 17% in close contacts.^3^^,^^4^ Although vaccines remain the cornerstone of prevention, during the influenza pandemic, their efficacy varied and supply could be delayed, highlighting the need for complementary strategies for influenza control and management.^5^ Previously, antiviral drugs for influenza, such as neuraminidase inhibitors (NAIs), have been used to alleviate the severity and duration of symptoms, but their effectiveness in reducing transmission remains uncertain.^6^ A novel antiviral drug, baloxavir marboxil (baloxavir) provided a paradigm shift, with trials demonstrating faster symptom relief and greater viral load reduction compared to NAIs.^7^^,^^8^

Recently, we read the study conducted by Monto et al. with great interest, which was the first randomized phase 3b trial to demonstrate that baloxavir can reduce influenza transmission in a real-world setting.^9^ The family clustering design of the study effectively reproduced the natural propagation dynamics, which was a significant improvement compared with previous community-based confounding variable studies. The study showed evidence that a single dose of oral baloxavir for early treatment of index patients can reduce the spread of influenza within households, making an important improvement in the influenza control and shifting the focus from postexposure prophylaxis and symptom relief to blocking virus transmission chain at the source.^9^

In this trial, early baloxavir treatment of index patients demonstrated a 29% reduction in household transmission of influenza within 5 days, consistent with the rapid inhibition effect of baloxavir on viral shedding.^9^ This significant virological effect may be related to its cap-dependent endonuclease inhibition, which can distinguish baloxavir from NAIs such as oseltamivir, the latter of which requires multiple days of administration and shows a slower reduction in viral load. However, the lack of statistical significance in symptomatic infections among contacts (5.8% vs. 7.6%) limited the clinical generalability of virological results. This might be due to the incidence rate in the placebo group being lower than expected, which likely reflect the behavioral changes caused by the COVID-19 pandemic (e.g., wearing masks and maintaining social distancing), potentially attenuated the therapeutic efficacy of baloxavir. Future trials are urgently needed to evaluate the effort of baloxavir in other settings. Meanwhile, more explorations are needed to evaluate the synergistic effect of baloxavir in vaccinated cohorts, as well as the elderly or those with weakened immune systems. Additionally, studies on the role of baloxavir in combination with prevention strategies, as well as its efficacy against emerging or pandemic influenza strains, are urgently needed.

Taken together, the findings from baloxavir trial are important for the development of public health interventions, particularly in multi-generational households and during the early stages of influenza outbreaks when vaccine coverage is incomplete. The study provides valuable guidance for influenza management in China, where multigenerational households are common and within-household transmission is a major driver of seasonal outbreaks. The close contact between family members can easily lead to the rapid spread of the virus, and the phenomenon of “one person gets sick, and the whole family is infected” is common. Importantly, this study extends the research perspective from “patient efficacy” to “family prevention and control”, addressing the previously unresolved clinical question of whether antiviral drugs can play an intervention role in the control of group transmission. Considering the relatively low influenza vaccination rate in China, these findings support the integration of rapid diagnostic testing and early antiviral therapy into epidemic prevention and control measures, especially during the peak seasons. Furthermore, the simplicity of single-dose oral administration may facilitate wider community adoption and reduce barriers to compliance.

To sum up, Monto and colleagues have provided us with valuable insights into the management of influenza. It showed strong evidence for reconsidering the strategic use of antivirals like baloxavir in controlling influenza—not only for treatment, but also for prevention. This dual efficacy is crucial for both seasonal influenza control and the prevention of future influenza pandemics.

## Contributors

Xu JF, Liu Y, and Zhang PP drafted the original manuscript. Xu JF conceived and edited the final version. Cao B contributed to writing review.

## Declaration of interests

None declared.
